# Treatment of comminuted metaphyseal distal femoral fractures with a micromotion-balancing osteosynthesis: an animal study

**DOI:** 10.1186/s12893-023-01939-2

**Published:** 2023-05-11

**Authors:** Zhengwei Duan, Hao Hu, Yang Wang, Diankai Wang, Hua Lu

**Affiliations:** 1 Department of Orthopaedics, Shanghai Tenth People’s Hospital, School of Medicine, Tongji University, Shanghai, 200092 China; 2 Department of Orthopaedics, Xinhua Hospital Affiliated to Shanghai Jiaotong University School of Medicine Chongming Branch, Shanghai, 202150 China

**Keywords:** Novel drill, Locking plate, Distal femoral fracture, Fracture healing, Animal experiments

## Abstract

**Background:**

Locking plates are commonly used in the treatment of comminuted metaphyseal distal femoral fractures. However, locking plates form a strong structure and promote asymmetrical callus formation, which is not conducive for rapid fracture healing and may increase fracture risk. To overcome this, we designed a micromotion-balancing fixation system based on locking plates.

**Methods:**

Six healthy pigs (Bama miniature pigs) were used to establish a model of bilateral comminuted distal femoral fracture (AO/ASIF: 33-C2). Standard drilling was performed on one of each pig’s hind limbs (control group), whereas eccentric drilling was performed on the other hind limb (experimental group). Both femurs were fixed with a 3-hole locking compression plate using 5-mm-diameter screws. At 12 postoperative weeks, all pigs were euthanized and the femurs with compression plates were radiographically examined. The level of fracture healing and loosening/internal fixation failure were recorded. Bone mineral density, number of trabeculae, trabecular morphology, and calcification precipitations were assessed.

**Results:**

All pigs survived, and the fractures healed. No complications related to fracture healing, such as infection and internal fixation failure, were noted. The bone mineral density of the near and far cortical calli, number of the near and far cortical callus trabeculae, and difference in bone mineral density between the near and far cortical calli in the experimental group were significantly higher than those in the control group (p < 0.01). However, the difference in the number of trabeculae between the near and far cortical calli was significantly lower in the experimental group than in the control group (p < 0.01).

**Conclusion:**

This newly designed system provides stable fixation for comminuted distal femoral fracture, increases the overall strain at the fracture site, and balances the strains at the near and far cortices to achieve uniform callus growth and fracture healing.

## Introduction

The incidence of distal femoral fractures (DFFs) increases with age [1]. Treatment options include external fixators, locking plates, and intramedullary nails. Cell deformation at the fracture site (strain) is key to healing. It also depends on the width of the fracture gap and the relative movement between fracture surfaces, which is influenced by the rigidity of the fixation structure. The overall structural rigidity can be modified using different implants, screw types, screw positions, and plates [2].

The locking plate is the most common implant in the surgical treatment of DFFs. It forms a fixed angle on each screw hole, with each screw head fixed to the plate through a locking mechanism and an overall rigidity that can be increased by stabilizing the screw angle [3]. Because the plate does not rely on friction at the plate interface to provide stability, it does not need to be in direct contact with the bone, which aids in protecting the blood supply of the periosteum [4]. This significantly improves the surgical outcomes for DFFs. Nonetheless, locking plates form a strong structure and promote asymmetrical callus formation, with a relatively larger callus at the far cortex (opposite side of the plate) than at the near cortex (plate side), which is not conducive to rapid fracture healing [5, 6]. Moreover, excessive rigidity and stress concentration increase the fracture risk around the internal fixation [7]. The length of the bridging zone could be increased by not using screws in holes that are close to the fracture site, thus reducing rigidity [8]; however, this technique has not been consistently proven to be effective [5].

Furthermore, excessive rigidity of the locking plate results in an unbalanced strain between the fracture ends and insufficient or uneven callus formation. To overcome these issues, far cortical locking (FCL) was developed [7], which provides varying degrees of micromotion at the fracture site, thereby creating a more physiological healing environment. It uses a screw with only partial threads, and the screw hole diameter on the near cortex is larger than the screw diameter. The delicate screw structure design can increase the strain and micromotion of the cortical bone on the plate side after screw locking. This makes the stress on both plate sides more uniform under axial load, which is conducive to symmetrical callus formation and fracture healing [9]. The overall stress of the FCL screws is evenly distributed along the screws, greatly reducing end screw fracture and re-fracture [10]. While the advantages of FCL have been confirmed both in vitro and in vivo [9, 13], issues regarding its capacity to withstand normal physiological load and maintain reliable stability when used in the treatment of osteoporosis patients and complications with its long-term use remain.

Additionally, how the parameters of the screw and screw hole can be controlled to achieve the required micromotion remains unknown [11]. The increase in the axial motion of the cortex on the plate side is limited and insufficient relative to that of the far cortex, resulting in an imbalance in cortical displacement on both ends of the fracture site, leading to asymmetrical callus growth [5]. Moreover, screw holes on the near cortex are round, causing unfavorable rotational and lateral stresses for callus formation at the fracture site. If the balance between structural rigidity and structural strength is lost, internal fixation fails, thereby making it costly and limiting its application.

To further increase the range of axial motion of the cortex on the plate side, compensate for the lack of FCL design, promote uniform displacement between fracture pieces, and induce symmetrical callus formation, we previously designed a micromotion-balancing fixation system. This system uses the conventional locking plate and screw system with newly designed drill bit and sleeves. After drilling, the screw hole diameter on the far cortex is the same as the diameter of the ordinary locking screw to achieve occlusal fixation between the locking screw and far cortex. The screw hole on the near cortex is oval because it has the same lateral diameter as that on the far cortex but its longitudinal diameter is larger. The longitudinal diameter increases with the length of the screw and the fracture gap; therefore, the proximal end of the screw can move in the screw hole of the near cortex, and the direction of movement is parallel to the long axis of the femur. When the femur is subjected to a force parallel to its long axis, the distal end of the screw is bent, the femur is moved downward, and the proximal end of the screw moves in the screw hole. In other words, the displacement of the far cortex is based on the screw bending during compression, while that of the near cortex is due to the screw hole being larger than the screw thread. Thus, the cortical micromotion on both sides of the fracture end is always closely balanced, thereby promoting symmetrical callus growth.

We sought to investigate the efficacy of our system in the treatment of comminuted metaphyseal DFFs in pigs to provide a reference for its clinical application. We hypothesized that our system would perform better at fracture healing than the standard system.

## Materials and methods

### Micromotion-balancing fixation system

In this study, the fracture gap was set at 1 cm (comminuted fracture). To achieve secondary healing, the strain at the far and near cortices should be controlled between 2 and 10% [12]. We selected 10% (1 mm) strain as the target value. A 5-mm-diameter screw was used. The length of the oval screw hole in the near cortex was 6 mm (1-mm strain plus 5-mm screw diameter), and the direction was parallel to the long axis of the femur.

**Fig. 1:**
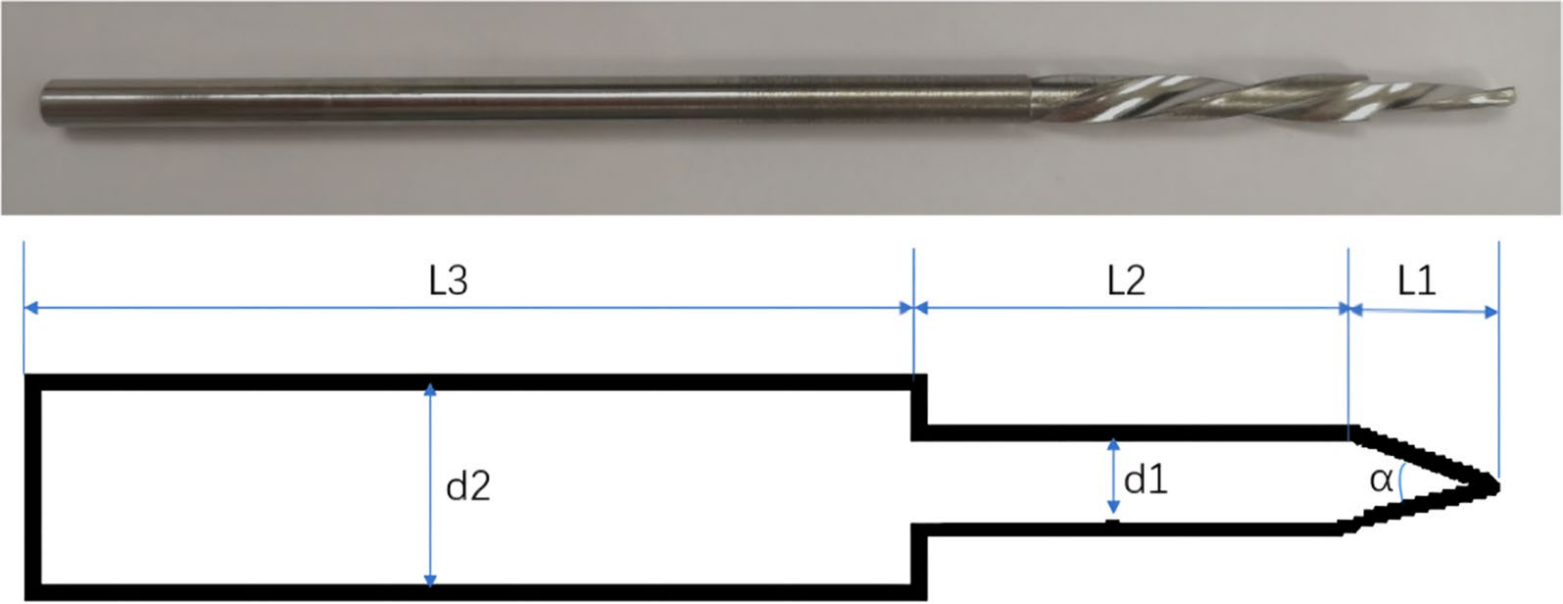
Real object picture and schematic diagram of the drill

**Fig. 2:**
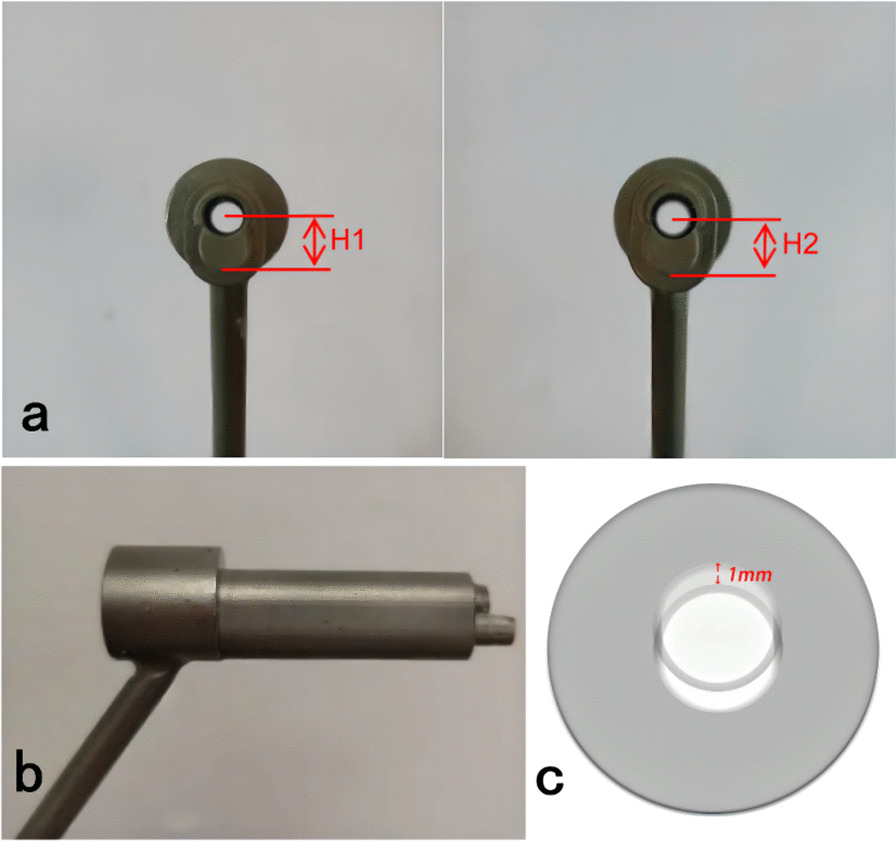
**a** Comparison of the standard (**a**) and newly developed sleeves. **b** Lateral view of the sleeves. **c** When the two sleeves overlap, the difference between them is 1 mm

**Fig. 3:**
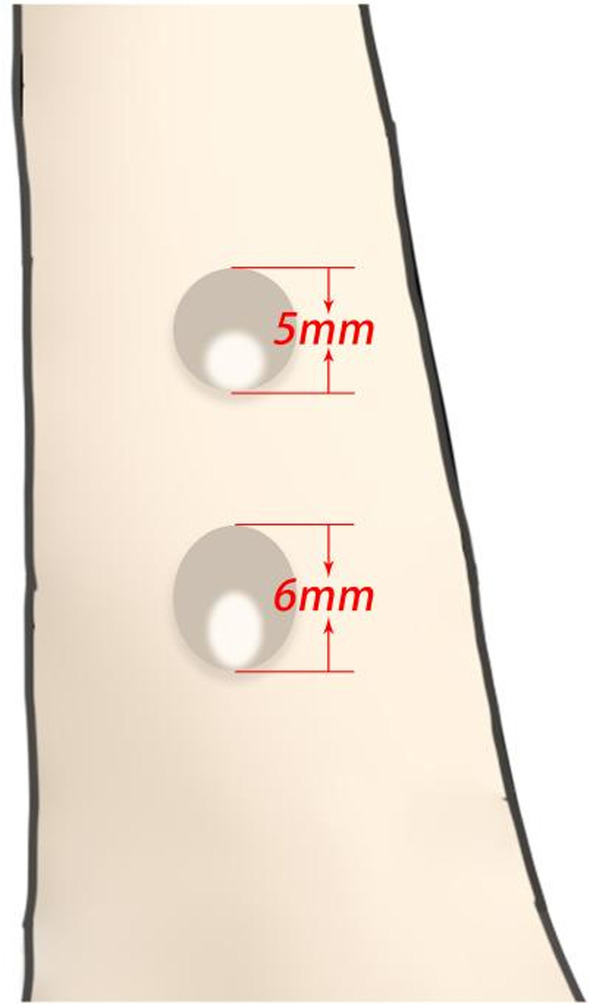
Comparison of the screw holes obtained with the standard and eccentric sleeves

The drill was divided into three parts: L1 (tip of the drill [2 mm]), L2 (first segment of the drill [the length should be less than the diameter of the distal femoral shaft [15 mm]), and L3 (second segment of the drill [100 mm]). Additionally, d1 is the diameter of the first segment of the drill (because a 5-mm screw was used in the study, the diameter of the drill was the same as that of the standard drill [i.e., 3.2 mm]), whereas d2 is the diameter of the second section drill (same as the screw diameter [i.e., 5 mm]) (Fig. 1). Two sleeves were designed: a standard sleeve, where the center of the sleeve hole was the same as the center of the hole in the locking plate, and a proximal eccentric sleeve. The eccentric distance can be controlled by the strain at the fracture site (10%). The eccentricity was 1 mm (difference between H1 and H2, with H being the distance from the boundary of the sleeve to the center) (Fig. 2a). A lateral view of the sleeve is shown in Fig. 2b, and the difference between the two sleeves when they overlap, which was 1 mm, is shown in Fig. 2c. When the standard sleeve and new drill were used, a 5-mm-diameter circular screw hole was obtained. Contrastingly, when the eccentric sleeve and new drill were used, a similar oval screw hole with a long diameter (6 mm) was obtained, and its direction was parallel to the long axis of the femur (Fig. 3).

**Fig. 4:**
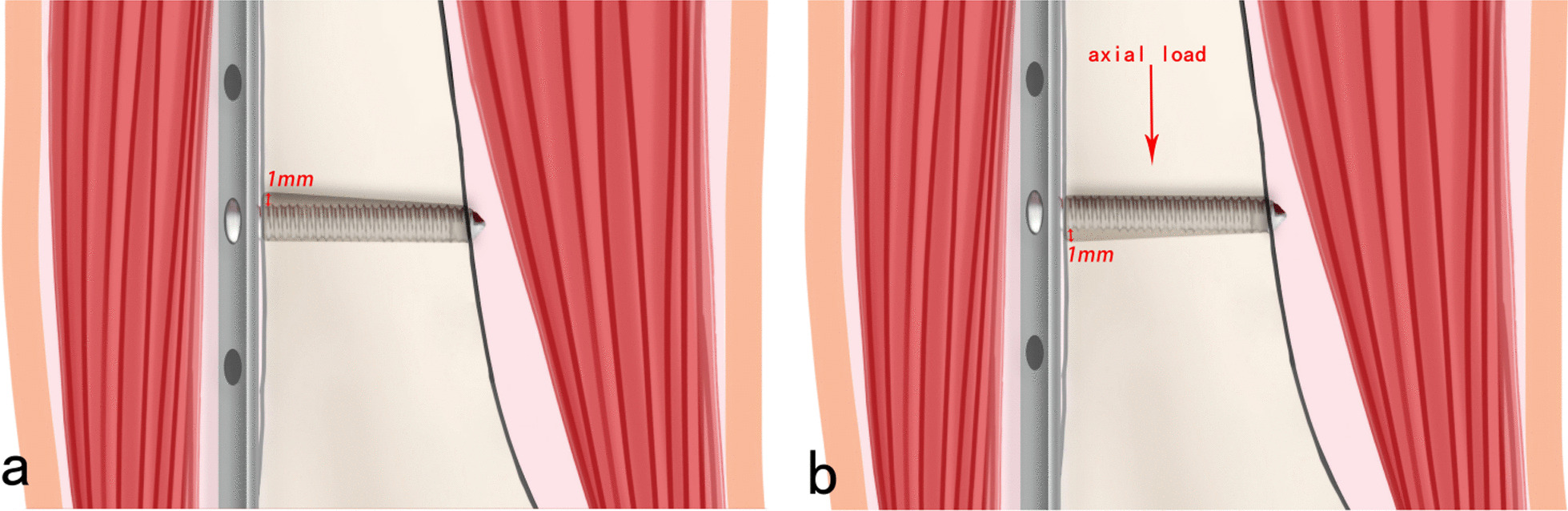
Procedure of the micromotion-balancing fixation system and force analysis

We drilled the locking screws into the oval and circular screw holes at the near and far cortices, respectively. Because the oval screw holes were larger by 1 mm than the screws, the proximal part of the screws could move within the screw holes (Fig. 4a). The diameter of the circular screw holes at the far cortex was the same as that of the screws to achieve occlusal fixation between the locking screws and far cortex. When the femur is subjected to a force parallel to its long axis, the distal end of the screw bends, the femur moves downward, and the proximal end of the screw moves in the screw hole, with a displacement of 1 mm, which can cause a strain of 1 mm between the fracture ends. Thus, the displacement of the far cortex was based on the bending of the screw under compression, whereas the displacement of the near cortex was due to the screw holes being larger than the screw diameter (Fig. 4b).

### Experimental animals and groups

Six experimental pigs (Bama miniature pigs), weighing 20–25 kg, and the sexes were randomized. were raised by the Animal Experimental Center of Xinhua Hospital, Shanghai Jiao Tong University School of Medicine, China. The exclusion criteria used when selecting the animals were obvious claudication; X-ray revealing obvious knee joint trauma or femoral deformity; and basic diseases, such as infection, tumor, or other metabolic system diseases. Random standard drilling was performed on one of each pig’s hind limbs (control group), whereas eccentric drilling was performed on the other hind limb (experimental group).

### Anesthesia, surgery and postoperative treatment

Each pig received an intramuscular injection of ketamine and midazolam, followed by inhalation anesthesia with a mixture of isoflurane and oxygen. When a sufficient level of anesthesia was achieved, animals were intubated and anesthesia was maintained with isoflurane mixed with oxygen. After endotracheal intubation, the animals were mechanically normoventilated with a positive end-expiratory pressure of 5 cmH_2_O. Fluid loss was substituted with continuous intravenous infusions of Ringer’s acetate and glucose solutions.

**Fig. 5:**
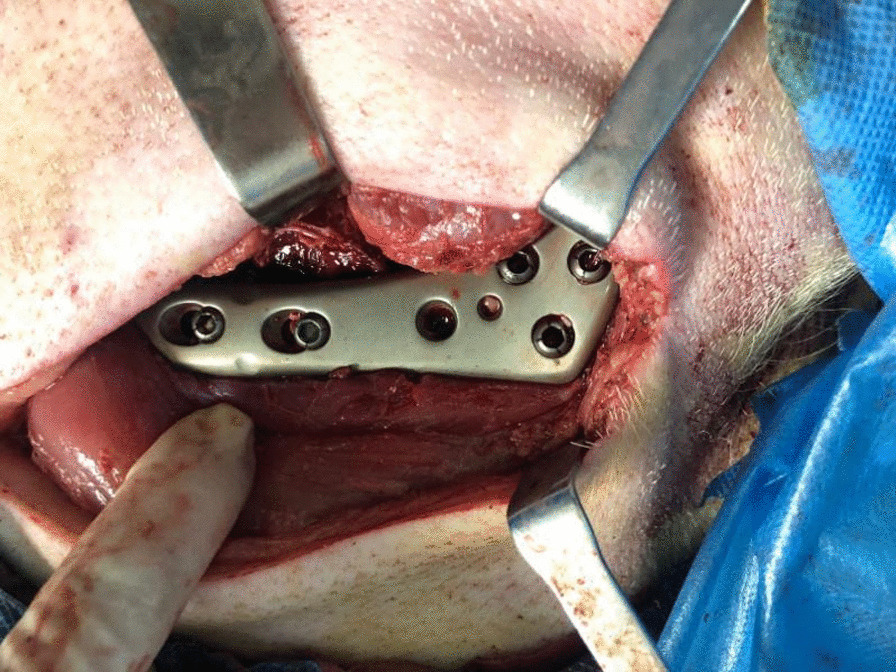
Establishment of the fracture models and placement of the plates

Each pig’s femur was operated on to construct a DFF model of a comminuted epiphyseal fracture (AO/ASIF:33-C2). Plates and screws were placed (Fig. 5) and the appropriate postoperative management was performed. At 12 postoperative weeks, the pigs were euthanized with fast intravenous injections of propofol (200 mg, as an overdose of anesthetics) and acute blood loss under anesthesia, the intact femurs were removed, and the femoral specimens were radiographically examined.

### Outcome evaluation

Femoral frontal X-rays were taken at 12 postoperative weeks and, together with femoral specimens, were used to assess fracture healing. The bone calli from plate side and opposite side (experimental and control) were taken, and the specimens were scanned using micro-CT (Skyscan 1076, Aartselaar, Belgium) to measure parameters, including bone mineral density (BMD) and number of trabeculae. Furthermore, histological observations were conducted through hematoxylin and eosin (HE) and von Kossa staining procedures.

### Statistical analysis

Data were processed using SPSS version 18.0 (IBM Corp., Armonk, NY, USA). The arithmetic mean values of micro-CT scan indexes (number of bone trabeculae and BMD) in both groups were calculated, and paired t-tests were performed. Statistical significance was set at p < 0.05.

## Results

### Postoperative situation

**Fig. 6:**
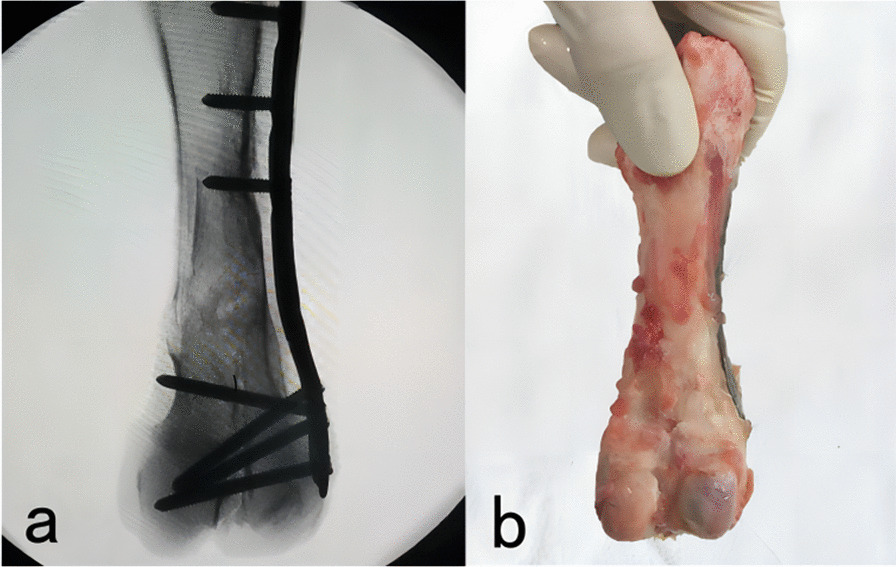
X-rays (**a**) and gross specimen status (**b**) at 12 postoperative weeks

All six experimental pigs survived and had good appetite and activity. Skin wounds showed good healing without any complications, such as infection or internal fixation failure. Radiographs obtained at 12 postoperative weeks showed callus formation, unclear medullary cavity of the distal femur, and restored continuity of the cortex (Fig. 6a). Gross specimens showed the disappearance of the osteotomy line in both groups and cortical bone-like callus formation (Fig. 6b).

### Micro-CT

**Fig. 7:**
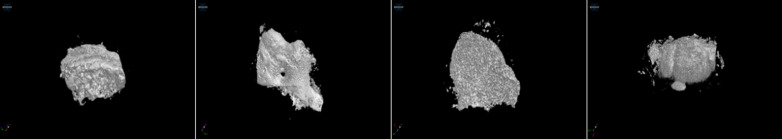
Micro-computed tomography three-dimensional images of callus specimens

**Fig. 8:**
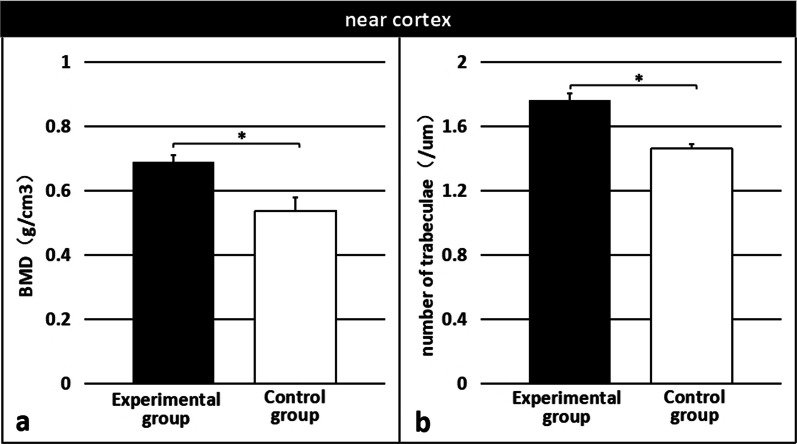
Bone mineral density (**a**) and the number of trabeculae (**b**) of the near cortical calli were significantly higher in the experimental group than in the control group. *Significant (p < 0.01)

**Fig. 9:**
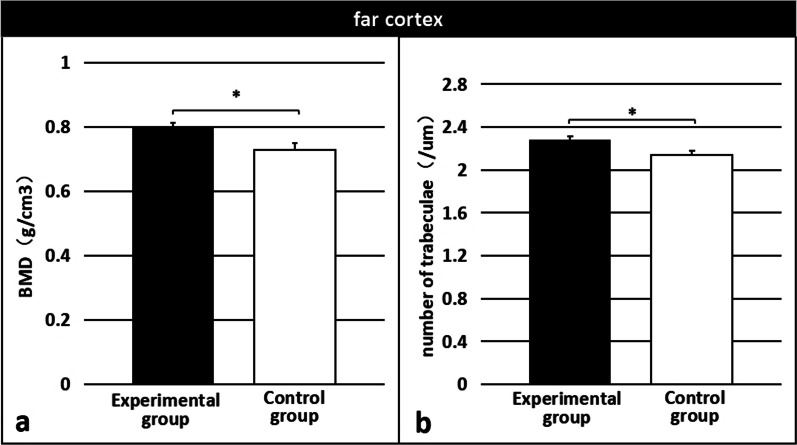
Bone mineral density (**a**) and the number of trabeculae (**b**) of the far cortical calli were significantly higher in the experimental group than in the control group. *Significant (p < 0.01)

Three-dimensional images of each specimen are shown in Fig. 7. Characteristic values were analyzed to obtain the BMD and number of trabeculae in each specimen. The results indicated that the BMD and number of trabeculae in both near and far cortical calli were significantly higher in the experimental group than in the control group (near: 0.6880 ± 0.022 g/cm^3^ vs. 0.5357 ± 0.043 g/cm^3^; p < 0.01 [Fig. 8a]; 1.763 ± 0.039/µm vs. 1.460 ± 0.031/µm; p < 0.01 [Fig. 8b] and far: 0.8011 ± 0.012 g/cm^3^ vs. 0.7295 ± 0.020 g/cm^3^, p < 0.01 [Fig. 9a]; 2.282 ± 0.027/µm vs. 2.141 ± 0.033/µm, p < 0.01 [Fig. 9b]).

**Fig. 10:**
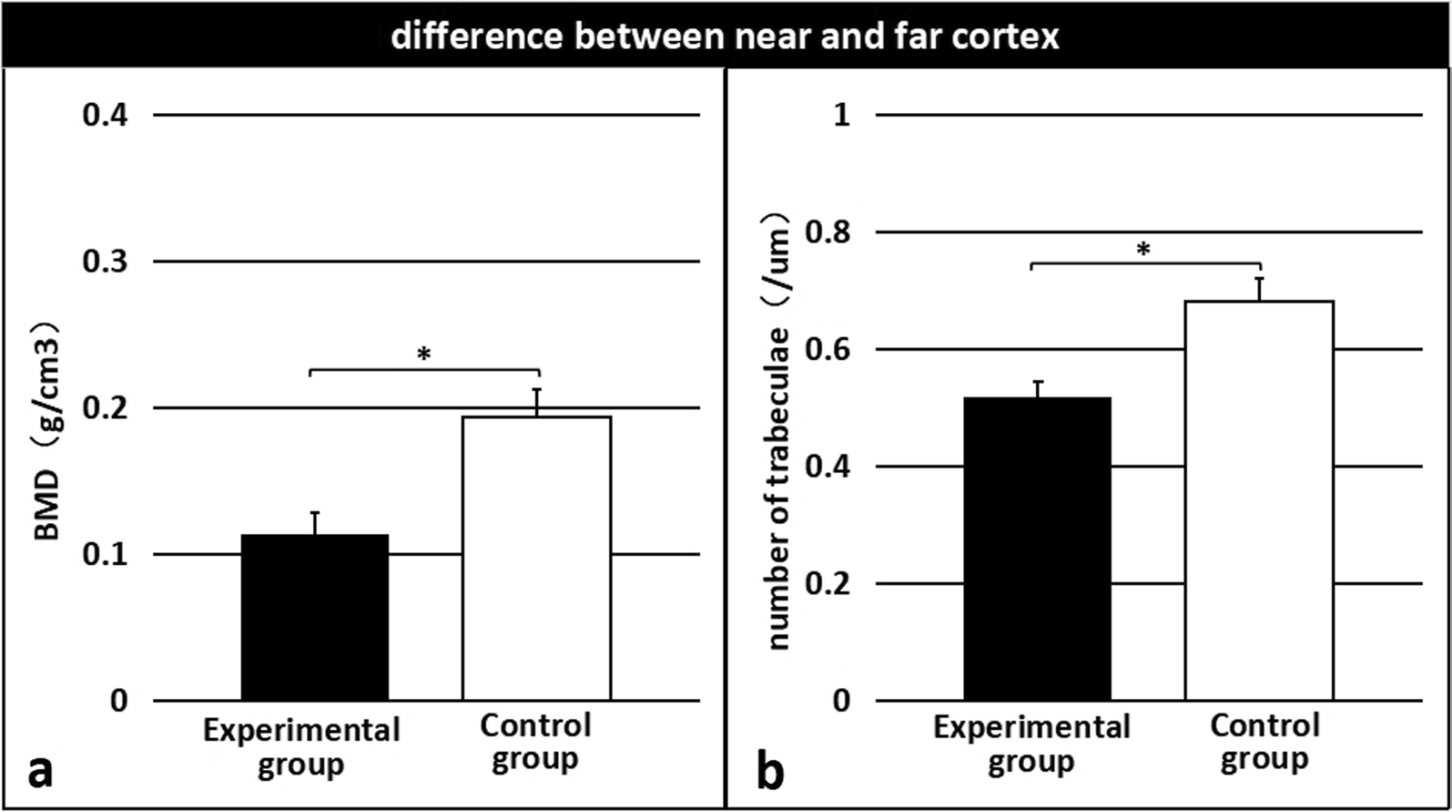
The difference in BMD (**a**) and the number of trabeculae (**b**) between the near and far cortical calli was significantly lower in the experimental group than in the control group. *Significant (p < 0.01)

The difference in BMD and number of trabeculae between the near and far cortical calli was significantly lower in the experimental group than in the control group (0.1131 ± 0.015 g/cm^3^ vs. 0.1938 ± 0.019 g/cm^3^, p < 0.01 [Fig. 10a]; 0.5186 ± 0.025/µm vs. 0.6806 ± 0.040/µm, p < 0.01 [Fig. 10b]).

### HE staining

**Fig. 11:**
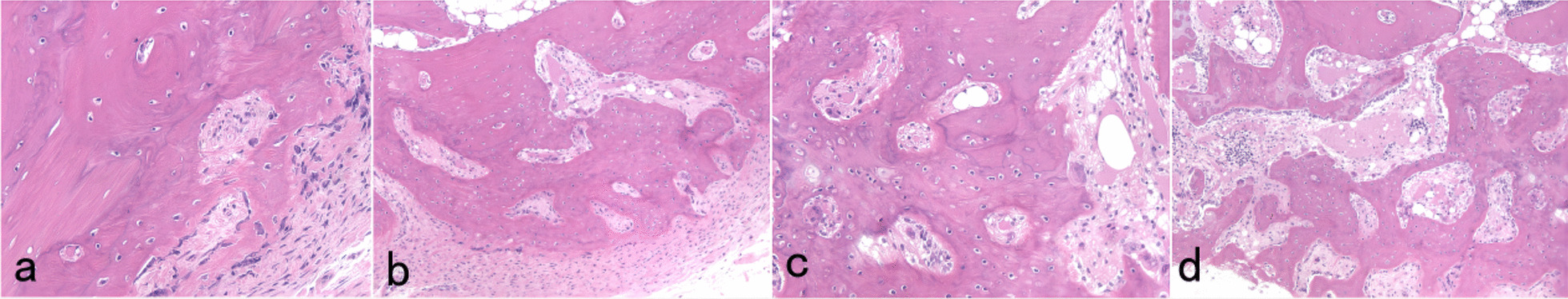
Hematoxylin and eosin staining of the experimental group (**a**, **b**) and control group (**c**, **d**). Magnification: × 200 in **a** and **c**, and × 100 in **b** and **d**

Staining results indicated bone tissue formation at the fracture site on the plate side in both groups, recanalized Haversian canals, and several osteoblasts (dark blue after HE staining). The experimental group had thicker trabeculae and higher numbers of trabeculae, osteoblasts, and bone-like tissues than the control group. Furthermore, the arrangement of bone trabeculae and Haversian canals was neat and close in the experimental group, with obvious vascularization (Fig. 11a, b). In the control group, the trabeculae and Haversian canals were relatively disorganized and sparse, and the number of osteoblasts was relatively small (Fig. 11c, d).

### Von Kossa staining

**Fig. 12:**
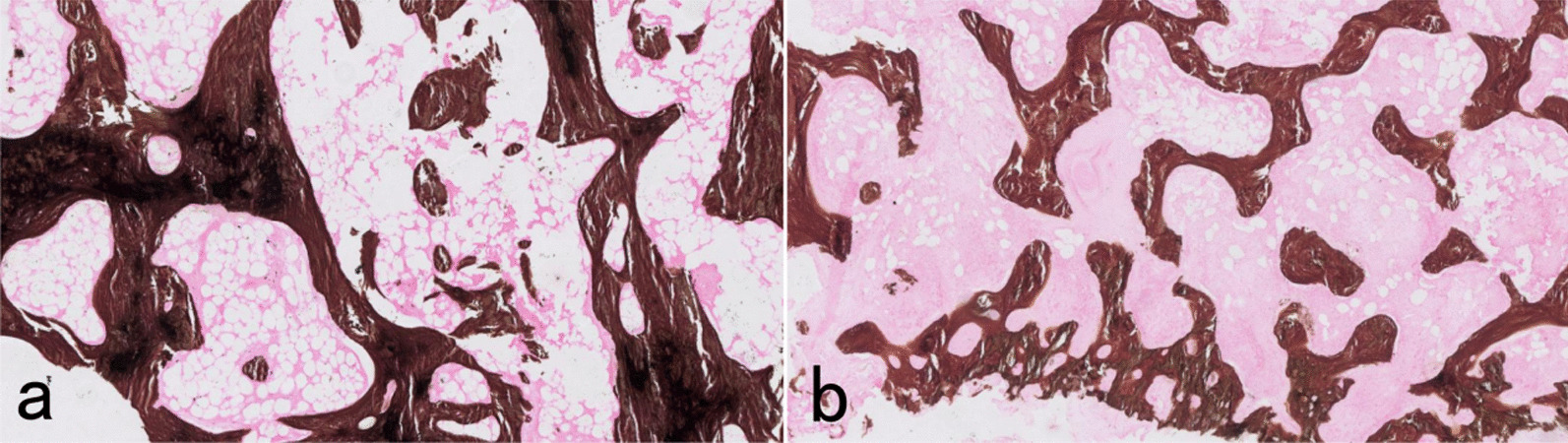
Von Kossa staining of the experimental group (**a**) and control group (**b**). Magnification: × 200

Staining results indicated dense and large calcium deposits (calcifications) in the callus specimens from the plate side in the experimental group (Fig. 12a), whereas those in the control group were sparsely distributed and had a small area (Fig. 12b).

## Discussion

Locking plates are widely used in the treatment of DFFs. However, they form an overly stable structure that results in asymmetrical callus formation, which affects fracture healing [5, 6]. Bottlang et al. [7] proposed the FCL technology, the advantages of which over locking plates have been confirmed both in vitro and in vivo [9, 13]; nevertheless, how the parameters of the FCL design can be controlled to achieve the required micromotion remains unknown. Furthermore, the displacement on both cortical sides of the fracture site remains imbalanced, resulting in a lack of symmetrical callus growth. The technology is also expensive and has few applications. Therefore, based on the idea that the strain at the far and near cortices could be controlled by adjusting the diameter of the drill bit and the eccentricity of the sleeves, we designed a novel micromotion-balancing fixation system that increases the displacement between the fracture ends and promotes uniform and symmetrical callus formation. The system still uses a locking plate but with anew drill bit and matching sleeves. The diameter and length of the drill can be determined according to the diameter of the screw and femur, while the eccentricity of the sleeves is determined according to the fracture gap. Previously, we tested our system in biomechanical experiments using artificial femurs (unpublished data). Here, investigated its efficacy in the treatment of comminuted metaphyseal DFFs in pigs to provide a reference for its clinical application. We hypothesized that our system would perform better than the standard system. At 12 postoperative weeks, the BMD and number of trabeculae of the calli at the near cortex were significantly higher in the experimental group than in the control group. In the experimental group, the bone callus on the plate side at the fracture site was composed mostly of lamellar bone; furthermore, the experimental group had thicker trabeculae and higher numbers of trabeculae, osteoblasts, and bone-like tissues than the control group. The trabeculae and Haversian canals in the experimental group were organized, compact, and with obvious vascularization, whereas those in the control group were disorganized and few, with a small number of osteoblasts. Furthermore, there were more calcification deposits on the plate side at the fracture site in the experimental group than in the control group, indicating that the experimental group performed better in terms of osteogenesis. Collectively, the results indicated that, compared with the traditional method, our system improved the biomechanical performance of the fracture site and was more conducive to DFF healing, thus confirming our hypothesis.

We expected that the strain at the far cortex of the fracture site would not significantly differ between the groups. However, we found that the BMD and number of trabeculae of the calli at the far cortex were significantly higher in the experimental group than in the control group, demonstrating that micromotion-balancing osteosynthesis can increase the overall strain at the fracture site. The difference in BMD and number of trabeculae between the calli of the near and far cortices was significantly smaller in the experimental group than in the control group, indicating that the system can help balance the strain at the near and far cortices, and promote uniform callus formation. Compared with the standard system, our system increased the strain at the near cortex, improved the overall structural rigidity, reduced the gap between the strains at the near and far cortices, and increased the load capacity. This will help patients with early weight-bearing exercise.

This study has several limitations. First, animal experiments cannot fully simulate the complex biomechanical environment of the human body, which limits the conclusions of the study. Second, due to the limited length of the animals’ femurs, we only used 3-hole locking plates, which reduced the grouping. Thus, the effects of different plate lengths and different numbers and distributions of screws on the biomechanics of fracture could not be compared. Nevertheless, our system solves the problem of asymmetrical callus formation, while ensuring stability.

## Conclusion

Our micromotion-balancing fixation system provides a simpler and more economic approach for the treatment of fractures. When an axial load is applied, displacement of the far cortex occurs due to the bending of the screw under compression, while displacement of the near cortex occurs due to the screw hole being larger than the diameter of the screw threads. This system provides stable fixation for comminuted distal femoral fracture, increases the overall strain at the fracture site, and balances the strains at the near and far cortices, thus promoting uniform callus growth and fracture healing.

## Data Availability

The datasets used and/or analyzed during the current study are available from the corresponding author on reasonable request.
